# Measuring Kidney Perfusion, pH, and Renal Clearance Consecutively Using MRI and Multispectral Optoacoustic Tomography

**DOI:** 10.1007/s11307-019-01429-z

**Published:** 2019-09-16

**Authors:** Atul S. Minhas, Jack Sharkey, Edward A. Randtke, Patricia Murray, Bettina Wilm, Mark D. Pagel, Harish Poptani

**Affiliations:** 1 Center for Pre-Clinical Imaging, Department of Cellular and Molecular Physiology, University of Liverpool, Crown Street, Liverpool, Merseyside UK; 2 School of Engineering, Macquarie University, Sydney, NSW Australia; 3 Department of Medical Imaging, University of Arizona, Tucson, AZ USA; 4 MD Anderson Cancer Center, Houston, TX USA

**Keywords:** acidoCEST, FAIR-EPI, pH, MSOT, Renal clearance rate

## Abstract

Purpose: To establish multi-modal imaging for the assessment of kidney pH, perfusion, and clearance rate using magnetic resonance imaging (MRI) and multispectral optoacoustic tomography (MSOT) in healthy mice. Kidney pH and perfusion values were measured on a pixel-by-pixel basis using the MRI acidoCEST and FAIR-EPI methods. Kidney filtration rate was measured by analyzing the renal clearance rate of IRdye 800 using MSOT. To test the effect of one imaging method on the other, a set of 3 animals were imaged with MSOT followed by MRI, and a second set of 3 animals were imaged with MRI followed by MSOT. In a subsequent study, the reproducibility of pH, perfusion, and renal clearance measurements were tested by imaging 4 animals twice, separated by 4 days. The contrast agents used for acidoCEST based pH measurements influenced the results of MSOT. Specifically, the exponential decay time from the kidney cortex, as measured by MSOT, was significantly altered when MRI was performed prior to MSOT. However, no significant difference in the cortex to pelvis area under the curve (AUC) was noted. When the order of experiments was reversed, no significant differences were noted in the pH or perfusion values. Reproducibility measurements demonstrated similar pH and cortex to pelvis AUC; however, perfusion values were significantly different with the cortex values being higher and the pelvic values being lower in the second imaging time. We demonstrate that using a combination of MRI and MSOT, physiological measurements of pH, blood flow, and clearance rates can be measured in the mouse kidney in the same imaging session.

## Introduction

The kidney is a complex organ that regulates waste elimination and systemic physiological conditions including pH. Waste elimination *via* the kidney involves filtration of blood in the glomerular capillary tuft that generates an ultrafiltrate of plasma, which is further purified during its transport along the tubuli of the nephron and also involves the proximal tubule, loop of Henle, and distal tubule [1, 2]. This transport can be characterized by a perfusion rate and filtration rate. Some or all of these processes may be impaired in patients with kidney injury or disease, culminating in progressive changes in pH, perfusion, and/or filtration. Therefore, non-invasive evaluations of pH, perfusion, and filtration have strong potential to assess kidney health and function.

Arterial spin labeling (ASL) magnetic resonance imaging (MRI) is a well-known method for measuring kidney perfusion rate in preclinical and clinical studies [3, 4]. In addition, chemical exchange saturation transfer magnetic resonance imaging (CEST MRI), or more specifically a version of this method known as acidoCEST MRI, has been used to measure pH in the kidneys of rodents with acute kidney injury, within preclinical tumor models, and within patients who have cancer [5–8]. To perform acidoCEST MRI, a contrast agent (CA) is injected and radiofrequency (RF) pulses are applied at the MR frequency of an exchangeable proton on the *CA.* A rapid exchange of the saturated proton on the CA with the near-by water protons causes a reduction in the water signal. The exchange rate between a CA and water is sensitive to pH, and therefore the CEST-based reduction in water signal is analyzed to measure pH. As the method involves acquisition of CEST MRI data before and after injection of the CA, static endogenous CEST contrast is eliminated by the subtraction of the two data sets. Moreover, the ratiometric procedure used for determining the pH value from acidoCEST MRI removes the potential dependence on concentration, T1 and T2 relaxation, and B1 inhomogeneity during the pH measurement and reduces the dependence on temperature to negligible levels in the physiological temperature range [8]. Other CEST MRI methods have also been developed to assess kidney physiology at the molecular and functional levels [9, 10].

Multispectral optoacoustic tomography (MSOT) is a novel imaging technique with the potential to measure renal excretory function in mouse models and has also been translated to the clinic [11–15]. It can resolve specific sources of light absorption such as a CA by exciting the subject with pulsed laser light in the near infrared (NIR) range, which causes thermoelastic expansion that generates a pressure wave, which is then detected with an ultrasound transducer. MSOT has previously been used to monitor kidney function in healthy mice and in mice with chronic kidney injury by measuring the clearance kinetics of IRDye-800 [16–18], referred to as IRDye henceforth. However, to our knowledge, both MSOT and MRI have not been used in conjunction to obtain renal pH, perfusion and renal clearance.

In this study, we sought to obtain a more comprehensive assessment of renal physiology in mice using MRI and MSOT. Specifically, we used acidoCEST MRI to measure renal pH, and ASL to measure renal perfusion in healthy mice. This was combined with MSOT assessment of IRDye clearance as a measure of renal clearance to demonstrate the feasibility of using these imaging modalities consecutively. Longitudinal studies were performed to investigate the reproducibility of MRI-based pH and perfusion measurements and MSOT-based renal clearance measurements.

## Materials and Methods

### *In Vitro* acidoCEST MRI Experiments

Chemical samples were prepared using clinical grade iopamidol (300 mg iodine/ml Niopam™ 300, Bracco UK Ltd.). The iopamidol was diluted with distilled water to 100 mM concentration and adjusted to pH 5.80, 6.11, 6.40, 6.63, 6.85, 7.05, and 7.30 using 200 mM hydrochloric acid. The pH was measured using a calibrated pH electrode (Mettler Toledo Inc., USA) while mixing the solution using a magnet-rotor assembly. Each solution was placed in a cylindrical 7-ml tube, the tube was fixed on a bed using tape, and the bed was placed inside the RF coil (PulseTeq Ltd., Surrey, UK). The samples were maintained at 33.5 ± 1.5 °C using a water heating system.

MRI studies were performed using a 9.4-Tesla (T) Biospec MRI scanner (Bruker Biospin, GmbH, Ettlingen, Germany) with a 27-mm diameter quadrature transceiver coil (Pulse Teq Ltd., Surrey, UK). A CEST MRI acquisition with fast imaging with steady-state precession (FISP) readout was developed using Bruker Paravision 6.0.1. The FISP acquisition used the following parameters: TR/TE = 3.0/1.5 ms, flip angle = 60°, in-plane resolution = 250 × 250 μm, slice thickness = 2 mm, field of view = 30 × 30 mm, axial orientation, linear encoding order, and unbalanced free induction decay (FID) mode. The CEST saturation block consisted of a series of RF pulses with the following parameters: flip angle = 180°, number of RF pulses in saturation block = 366, saturation time = 5.0 s, saturation power = 2 μT. A series of 60 frequencies were saturated to acquire a CEST spectrum with 4 frequencies of 1-ppm increments from + 10 to + 7 ppm, 46 frequencies of 0.1978 ppm increments from + 6 to − 2.9 ppm, 8 frequencies of 1 ppm increments from − 3 to − 10 ppm, and two frequencies at ± 100 ppm (to measure MR signal at saturation effects far from water). To increase the signal-to-noise (SNR) ratio, six averages were used for each of the saturation frequencies.

### CEST-pH Calibration

The CEST-FISP images from the seven samples of iopamidol and different pH values and one sample of distilled water were segmented from background, and the mean signal at each saturation frequency was measured to create a CEST spectrum for each sample. Each CEST spectrum was smoothed with a spline function using MATLAB 2018b (Mathworks Inc., USA). After smoothing, the signal from the distilled water sample was subtracted from the signal from iopamidol samples at different pH values. This difference was fitted to a sum of two Lorentzian line shapes with peaks at 4.2 ppm and 5.6 ppm. A log_10_ ratio of CEST peaks at 4.2 ppm and 5.6 ppm was calculated from the amplitudes of the fitted lines. This experimental CEST ratio was regressed against the pH of each sample measured using a pH meter, and the regression coefficients were calculated to form a linear calibration curve.

### MRI-MSOT for Imaging Mouse Kidney

Male Balb/c mice (*N* = 10) were purchased from Charles River, UK, and were housed with *ad libitum* access to food and water. Mice were between 6 and 8 weeks old weighing 20–25 g at the time of imaging. All MRI-MSOT experiments were performed in the same anesthesia session. All animal experiments were performed under a license granted under the Animals (Scientific Procedures) Act 1986 and were approved by the University of Liverpool ethics committee. Experiments are reported in line with the ARRIVE guidelines.

For each MRI-MSOT experiment, animals were anesthetized using isoflurane and fur was removed from the torso through shaving and depilatory cream (Veet hair removal, sensitive skin cream, with aloe vera). A 0.28-/0.61-mm inner/outer diameter (ID/OD) fine bore polythene tube was inserted into the tail vein to serve as a catheter to administer the IRDye (Licor, UK), during MSOT and iopamidol during MRI.

In the first experiment (*N* = 3), the tail vein catheterized animal was placed on a respiration pillow, and a temperature probe was inserted rectally. The animal was fixed on a bed using adhesive tape and the bed was inserted inside a 27-mm diameter quadrature transceiver coil (PulseTeq Ltd.). During the MRI experiment, the temperature was maintained at 34.5 ± 1.5 °C using a water heating system. The respiration rate and core body temperature of the animal were continuously monitored during the experiment (SA Instruments, Inc., USA). After MR imaging, the animal was removed from the bed and transferred to the MSOT scanner (iThera Medical, Munich, Germany) using an anesthetic trolley, with a time gap of approximately 3 min. The animal was allowed to equilibrate for 15 min at 34 °C before MSOT imaging as described below. To test whether IRDye affects MR based measurements of pH and perfusion the above process was repeated in the reverse order, with MRI following the MSOT measurements.

### Reproducibility of MRI-MSOT Experiments for *In Vivo* Mouse Kidney Imaging

To test the reproducibility of the MRI and MSOT studies in mice (*N* = 4), experiments on the same animal were performed on day 1 and day 4 where MRI was performed first followed by MSOT. The experiments were performed as described above.

### MRI Procedure

The *in vivo* MRI experiments were conducted using a 9.4-T Bruker Biospec MRI scanner. A localizer sequence was used to locate kidneys in the axial plane. A 2-mm-thick axial slice was chosen through the center of kidney and FAIR-EPI data were acquired for perfusion imaging. The scan parameters were TR/TE = 12,000/16.82 ms; inversion time = [26, 400, 800, 1200, 1600, 2000, 2400, 2800, 3200, 3600, 4000, 4400, 4800, 5200, 5600, 6000] ms. This was followed by the same CEST-FISP protocol that was used for the *in vitro* experiments. The first set of data were acquired without iopamidol injection. After the first scan, 200 μl of iopamidol was slowly administered *via* the catheter between 120 and 180 s and six sets of CEST-FISP data were acquired immediately after the injection. The FISP data acquisition used the following parameters: TR/TE = 3.26/1.63 ms; flip angle = 60°; in-plane resolution = 234.4 × 234.4 μm; slice thickness = 2 mm; field of view = 30 × 30 mm; linear encoding order; unbalanced FID mode. Respiratory gating was used to minimize motion artifacts. The total scan time with respiration gating was approximately 1 h.

Regions of interest (ROI) were drawn to segment the left and right kidneys. The 6 post-injection images at each saturation frequency were averaged to improve signal-to-noise. The first FISP data without the iopamidol injection were subtracted from the post-injection, averaged data to remove endogenous CEST signals and the direct saturation of water. A CEST spectrum was obtained for each pixel in the ROI. The pH for each pixel in the left and right kidney was calculated using the calibration curve obtained from the chemical sample experiments.

### MSOT Procedure

MSOT studies were performed using an InVision 256-TF MSOT imaging system (iThera Medical, Munich, Germany). Imaging was focused primarily on the right kidney as the spleen absorbs strongly in the near infrared range and can influence measurements in the left kidney. However, wherever possible (*n* = 6), the data from the left kidney was also processed, with an acquisition rate of 10 frames per second and averaging 10 frames to minimize breathing artifacts, and with wavelengths of 775 nm and 850 nm. Data sets were recorded for 3 min, followed by administration of 20 nmol IRDye which was administered in 200 μl in 10 s. Data sets were reconstructed using a model linear algorithm and a difference protocol (775 nm–850 nm) for IRDye imaging (View MSOT software). ROIs were drawn around the cortex and the renal pelvis/medulla of the kidneys to measure mean pixel intensity and the area under the curve for cortex:pelvis (C:P AUC) and exponential decay times, were calculated using Origin software (OriginLab Corporation, USA).

### Statistics

Microsoft Excel was used to compute the mean and standard deviation for all the measured values. Two-tail *t* test was performed to compute the *p* values. Coefficients of variation were calculated using the following equation: Standard deviation / Mean = Coefficient of variation, indicating the extent of variation in relation to the mean of the group. The Pearson correlation coefficient between the pH values of cortex and pelvis was calculated using Microsoft Excel.

## Results

**Fig. 1.**
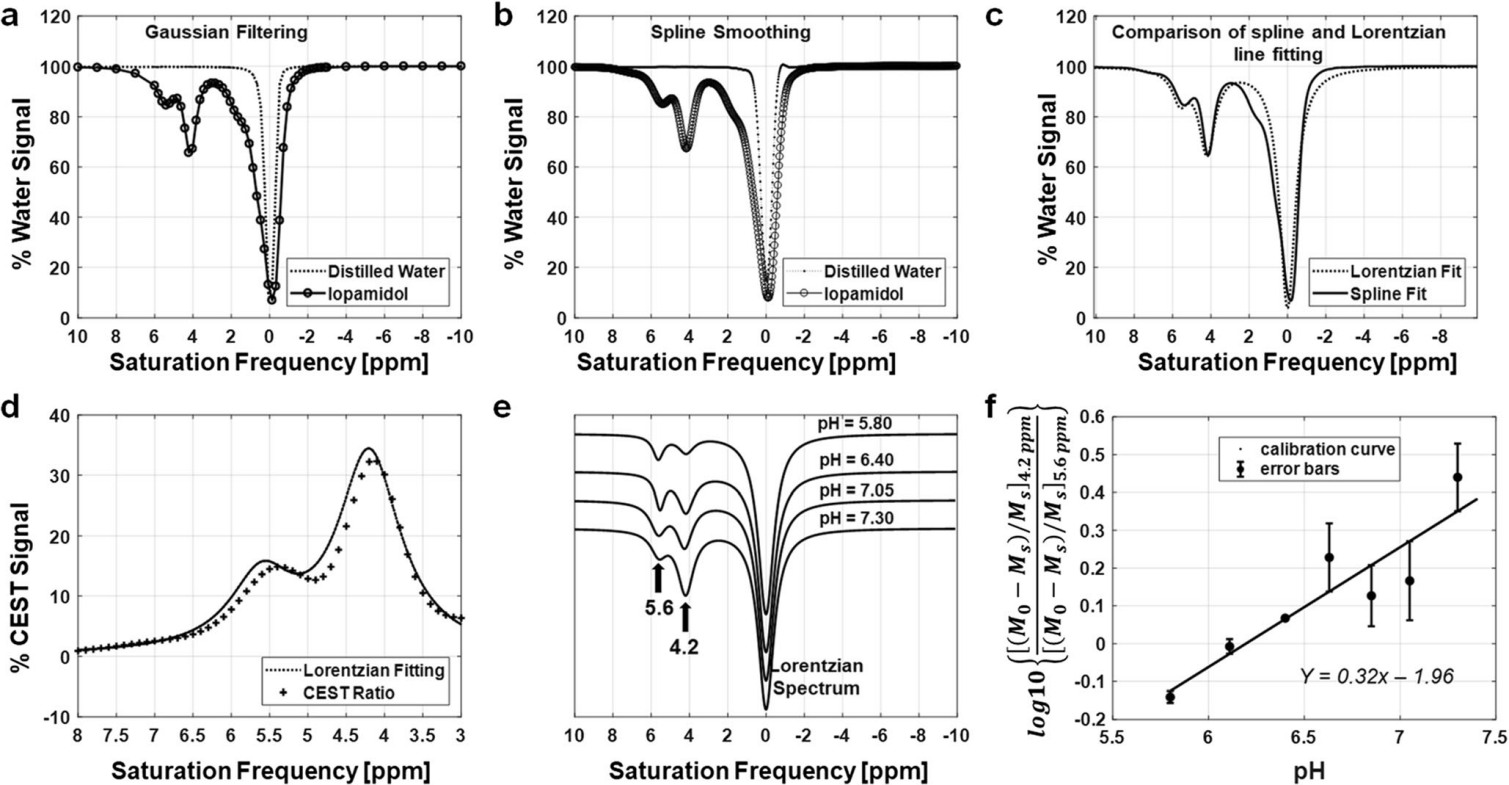
Data processing steps to obtain a pH calibration curve. **a**, **b** The percentage water signal over different saturation frequencies, obtained from chemical samples made of distilled water and iopamidol: **a** the Gaussian filtered signal; and **b** spline filtered signal. **c** The percentage water signal from iopamidol for Lorentzian line shapes fitted to the data in **b**. A spline fitted curve is also shown in **c** for comparison with Lorentzian fitted curve. **d** The percentage difference signal (CEST ratio) between distilled water and iopamidol. **e** The Lorentzian spectra over different pH values. **f** The pH calibration curve based on a log_10_ ratio of the two CEST signal amplitudes and the corresponding error bars.

A CEST-pH calibration curve was generated from chemical sample data (Fig. 1a–f). Comparisons of processed signals from samples of distilled water and iopamidol revealed peaks in CEST spectra at 0, 4.2, and 5.6 ppm (Fig. 1a–c). The Lorentzian line shape fitting demonstrated close agreement with the experimental, processed CEST spectra (Fig. 1d). Notably, a minor systematic difference between the Lorentzian line shape fitting and the CEST spectra was attributed to a broader linewidth for the direct saturation of water in samples that had iopamidol, relative to the control sample without iopamidol due to T2-exchange relaxation caused by iopamidol. However, this minor systematic difference affected both CEST signals, so that this minor error was effectively canceled by our ratiometric analysis. The CEST ratio increased linearly with pH (Fig. 1e), resulting in a linear pH calibration curve *y* = 0.32*x* − 1.96 (Fig. 1f).

**Fig. 2.**
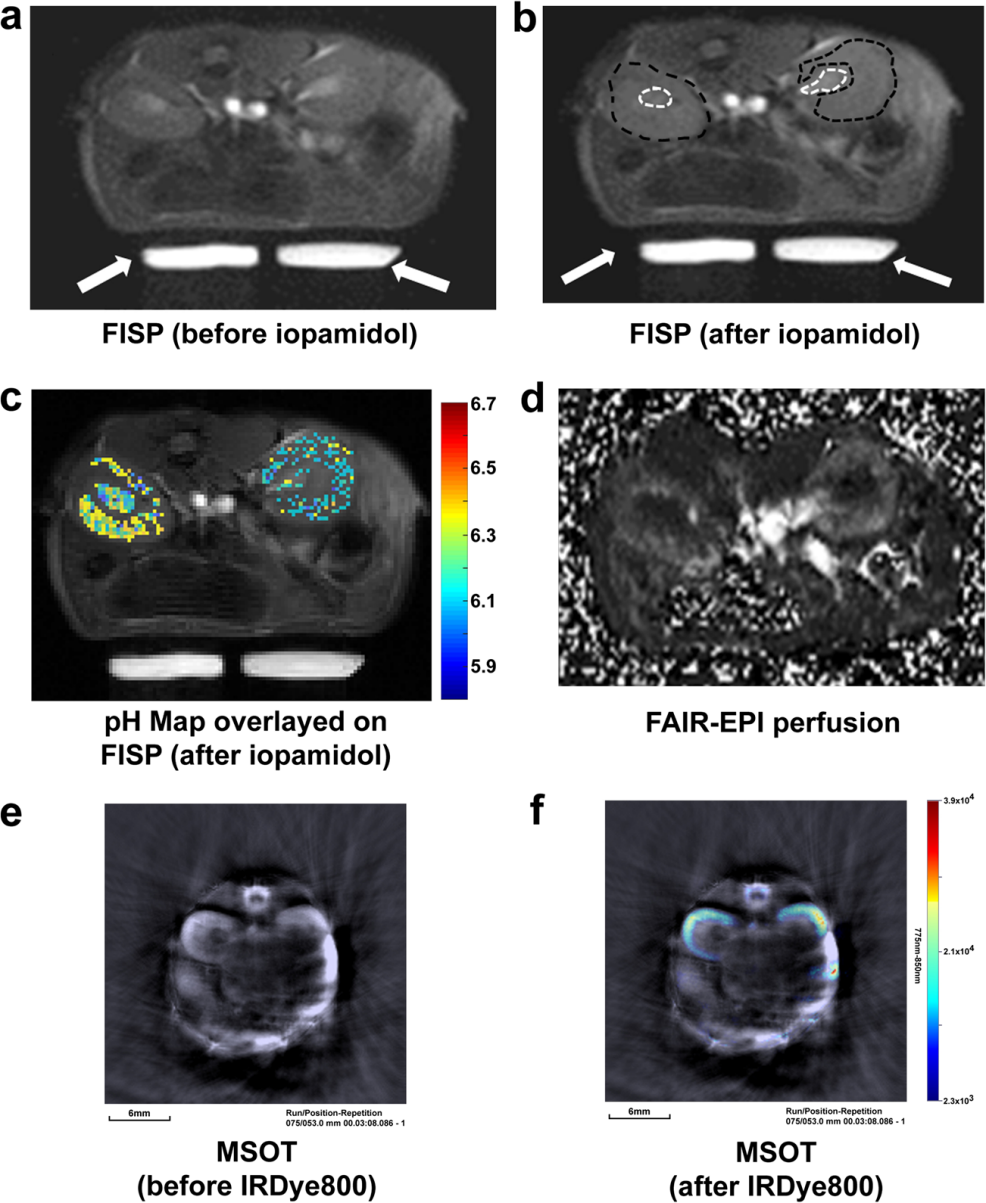
Typical MRI and MSOT images from a mouse in the axial plane. The FISP images **a** before and **b** after iopamidol injection. The dotted black and white curves in the left and right kidneys show the region-of-interest in cortex and pelvis, respectively. White arrows show the location of warm water tubes circulated in the mouse bed to maintain body temperature. **c** The pH map overlayed over the left and right kidney. **d** The perfusion image obtained using FAIR-EPI. The MSOT images **e** without and **f** with IRDye. The scale bar in the lower left corner of the MSOT images reflects 6 mm in length.

The results obtained from acidoCEST MRI (Fig. 2a, b) were used to generate a pH map (Fig. 2c) with a pH range from 5.8 to 6.7. This range was within the range of pH 5.8 to 7.3 used to generate the CEST-pH calibration. In Fig. 2c, 44 % of pixels in the cortex and 72 % of pixels in the pelvis region were successfully evaluated (when data from both the left and right kidneys were combined) with acidoCEST MRI. In Fig. 6a and b, where a comparison between day 1 and day 4 pH values is shown, 37 ± 20 % of pixels in the cortex and 56 ± 26 % of pixels in the pelvis were successfully evaluated with acidoCEST MRI. This incomplete coverage attests to insensitivity of *in vivo* CEST MRI, and our conservative approach to analysis that ensures that only reliable pH values are reported.

A FAIR-EPI pulse sequence was used to generate a perfusion map, which clearly demonstrated differences in blood flow between the kidney cortex and pelvic regions (Fig. 2d). Average kidney perfusion values for seven animals from the studies where MRI was performed prior to MSOT were 108 ± 30 ml/100 g/min for cortex and 6 ± 3 ml/100 g/min for pelvis. Subsequently acquired MSOT images before and immediately after IRDye administration revealed the distribution of the dye in the cortex (Fig. 2f). These experiments demonstrated that both MR and MSOT imaging allowed visualization of both kidneys, specifically with respect to differences between pelvic and cortex regions.

### The Effect of the Order of the Imaging Scans

**Fig. 3.**
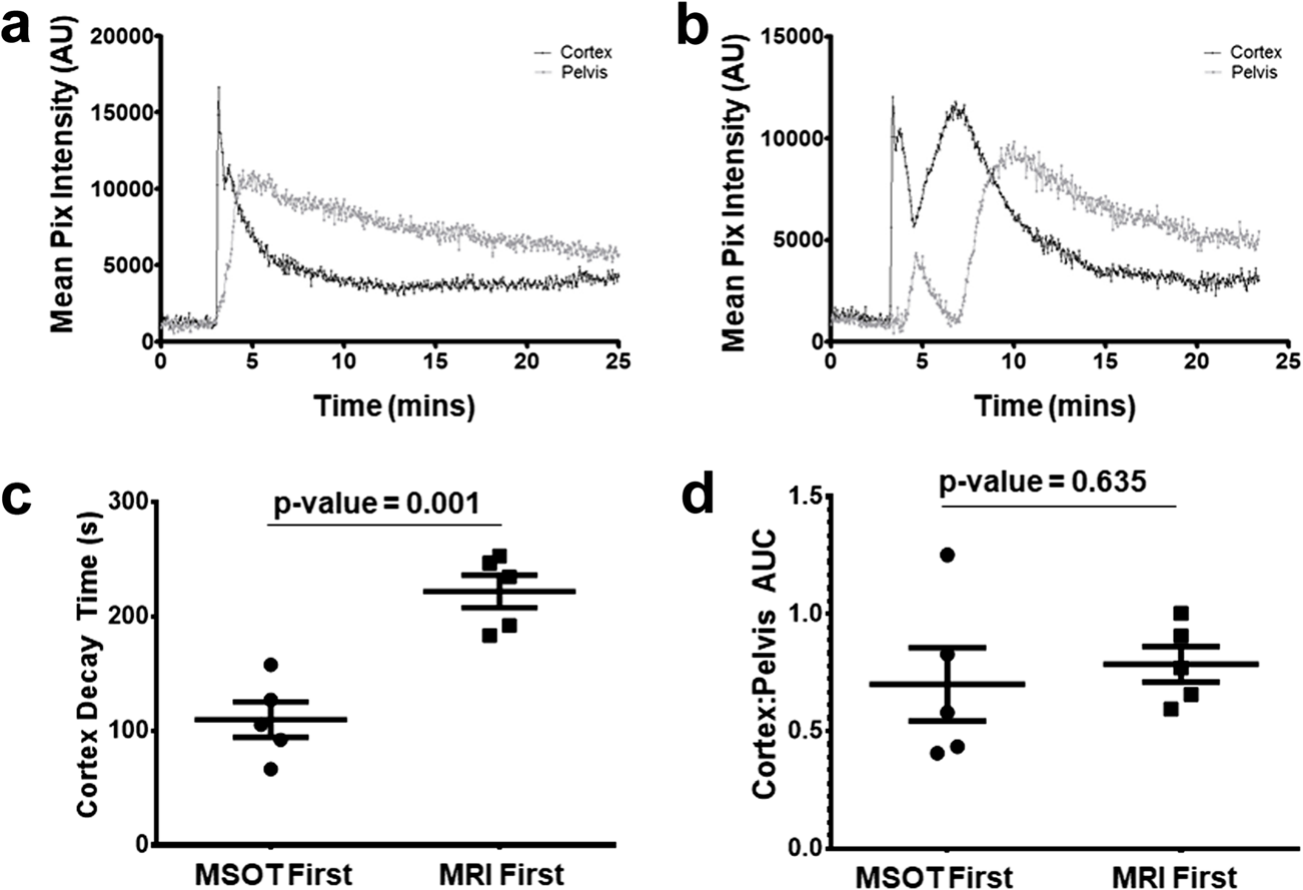
Clearance kinetics of IRDye in the cortex and renal pelvis/medulla of healthy Balb/c mice. Mice received 20 nmol of IRDye through a tail vein catheter over 10 s. **a** The temporal changes in mean pixel intensity in the cortex and renal pelvis show the kinetics of IRDye in a typical mouse that had not received iopamidol prior to receiving IRDye. **b** The kinetics of IRDye in mice which received iopamidol prior to receiving IRDye. The clearance kinetics of IRDye from mice that received either IRDye prior to receiving iopamidol (MSOT first) and mice that received iopamidol prior to receiving IRDye (MRI first), as measured with the **c** cortex decay time and **d** the C:P AUC. Error bars represent the standard error.

The relatively higher viscosity of the iodinated CT contrast agents [10, 19] used in acidoCEST can influence kidney clearance. We assessed the influence of iopamidol on MSOT measurements by comparing the IRDye clearance kinetics in mice before or after administration of iopamidol. The typical IRDye clearance kinetics showed one clear peak, with subsequent decay in both the renal cortex and pelvic regions of a healthy mouse (Fig. 3a). However, after iopamidol administration and MR imaging, the IRDye clearance kinetics revealed altered dynamics, including several peaks both in cortex and pelvis (Fig. 3b), and this phenomenon was observed in all the animals. Further analysis demonstrated that the IRDye cortex decay time was significantly elevated (*p* = 0.001) in mice that received iopamidol for MR imaging before IRDye when compared with mice that received iopamidol after IRDye clearance measurements (Fig. 3c). By contrast, the C:P AUC of IRDye clearance (Fig. 3d) was not significantly different whether MRI was carried out prior to MSOT or *vice versa*.

**Fig. 4.**
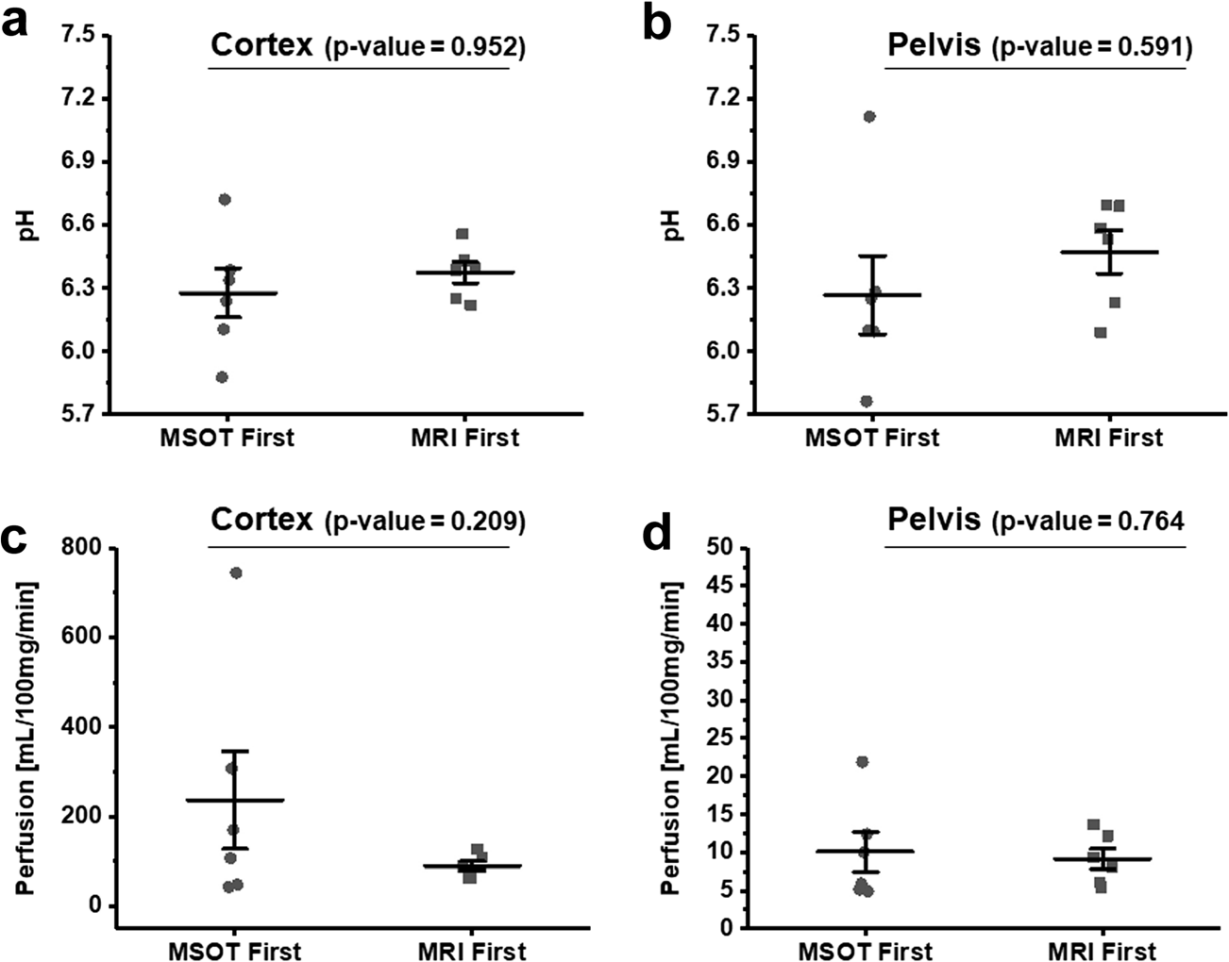
Comparisons of pH and perfusion values from mice which received either IRDye prior to receiving iopamidol (MSOT first) and mice that received iopamidol prior to receiving IRDye (MRI first). The pH in **a** cortex and **b** pelvis and perfusion in **c** cortex and **d** pelvis, respectively. Each data point represents an individual kidney. Error bars represent the standard error.

We then examined whether the administration of IRDye impacted MRI measurements. Both cortical (Fig. 4a, c) and pelvic (Fig. 4b, d) pH and perfusion measurements using MRI, carried out either before or after IRDye injection, demonstrated no significant differences in the parameters between the two groups, indicating that the MSOT contrast agent had negligible effect on the acidoCEST and perfusion MRI parameters. However, performing MSOT prior to MRI increased the variability of the perfusion and pH measurements as demonstrated in the mean, standard deviation, and coefficients of variation values (Table 1). The pH values over the whole population for the left and right kidney cortex and pelvis were 6.4 ± 0.3 and 6.4 ± 0.4, respectively (Fig. 4a, b). In general, a good correlation (0.8) was noted between the pH of the kidney cortex and the pelvis.

**Table 1.** Mean and standard deviation of MRI perfusion and pH measurements (with coefficients of variation in brackets), when carried out prior to and after MSOT measurements.

	Cortex perfusion (ml/100 g/min)	Pelvis perfusion (ml/100 g/min)	Cortex pH	Pelvis pH
MSOT first	237 ± 244 (1.03)	10 ± 6 (0.60)	6.28 ± 0.26 (0.04)	6.27 ± 0.41 (0.06)
MRI first	90 ± 23 (0.26)	9 ± 3 (0.33)	6.29 ± 0.19 (0.03)	6.39 ± 0.30 (0.05)

### Reproducibility of MSOT Kidney Filtration, MRI Perfusion, and pH Measurements

**Fig. 5.**
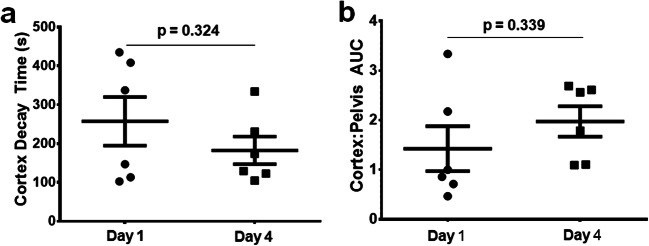
Comparisons between the renal clearance kinetics as measured by MSOT between days 1 and 4 in healthy Balb/c mice. Each data point represents an individual mouse kidney. **a** Cortex decay time and **b** C:P AUC values. Error bars represent the standard error.

**Fig. 6.**
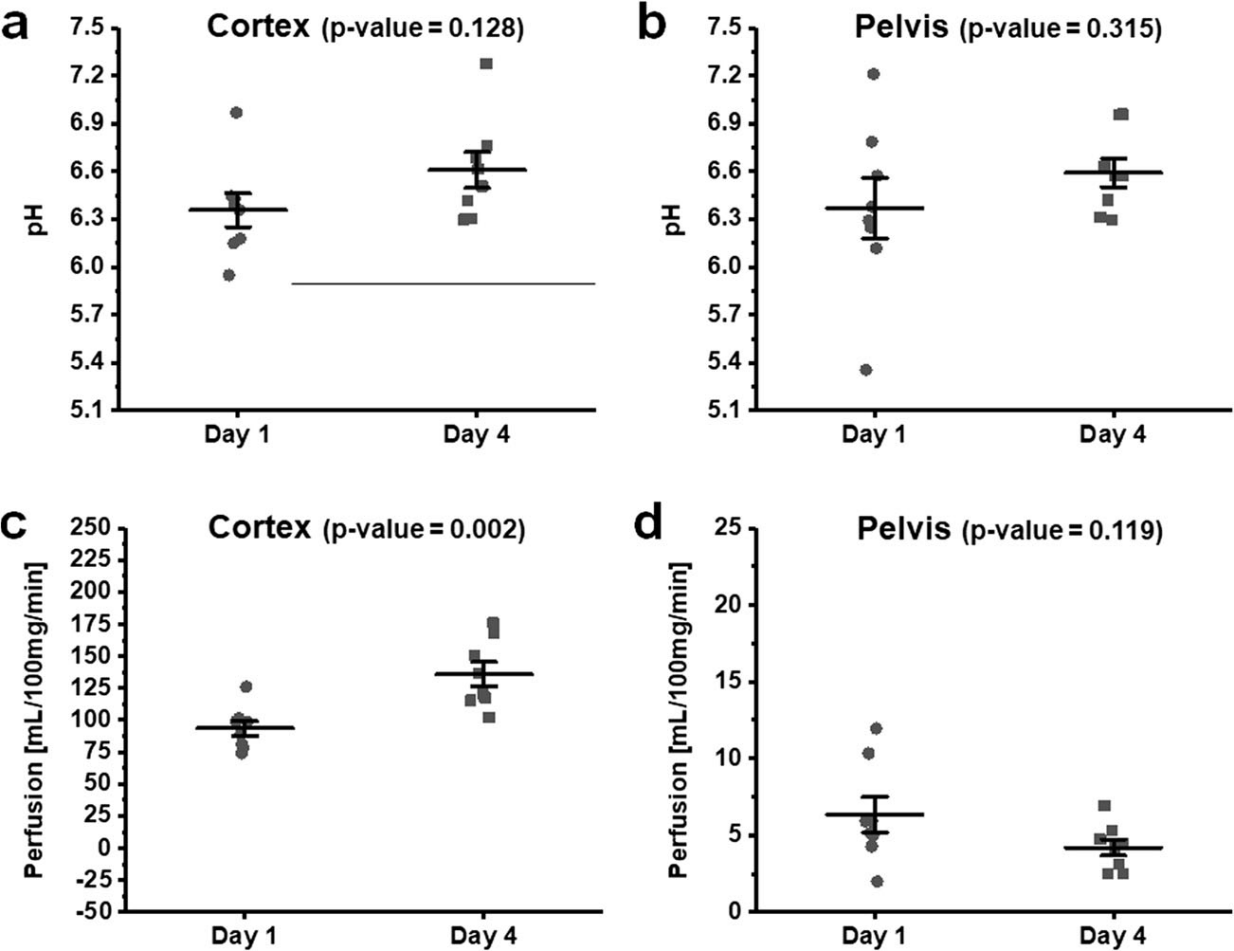
Comparisons of pH and perfusion values on day 1 and day 4 from mice which received iopamidol prior to receiving IRDye (MRI first). The pH in **a** cortex and **b** pelvis, and perfusion in **c** cortex and **d** pelvis, respectively. Each data point represents an individual kidney. Error bars represent the standard error.

To determine whether IRDye clearance by MSOT was reproducible over multiple days, the IRDye cortex decay time (Fig. 5a) and C:P AUC (Fig. 5b) were assessed on day 1 and day 4 in the same mouse. MRI measurements were carried out first, followed by MSOT measurements. No significant differences in the IRDye cortex decay time or the C:P AUC were observed between the two assessment dates. We also examined the reproducibility of the MRI determined perfusion and pH measurements between days 1 and 4 in both the cortex (Fig. 6a, c) and pelvic regions (Fig. 6b, d) of the kidney. There were no significant differences in the pH measurements in either the cortex or the pelvic regions of healthy mice. However, there was a significant increase in perfusion in the cortex (*p* = 0.002, Fig. 6c) with a concomitant non-significant decrease in the pelvic region (*p* = 0.119, Fig. 6d) of the kidneys between days 1 and 4.

## Discussion

This study established a method for analyzing renal clearance using MSOT, renal perfusion and pH using MRI in the same anesthesia session. To our knowledge, this is the first investigation that has combined these methods for assessing kidney function.

The CEST-based pH measurement is dependent on the accuracy of the CEST-pH calibration. The CEST signal at 5.6 ppm becomes weak at high pH due to base-catalyzed exchange that is too fast for the CEST mechanism. For comparison, the CEST signal at 5.6 ppm becomes very weak at pH 6.8 and 7.0 (and higher pH values) at 3 T and 7 T magnetic field strengths [20]. The present study at 9.4 T shows that the CEST peak at 5.6 ppm is still detectable at pH 7.3 (Fig. 1e). This is intuitive, because the greater chemical shift values in Hz at 9.4 T allow the proton at 5.6 ppm to have a higher chemical exchange rate and still generate CEST relative to the chemical shifts in Hz units that are typically observed at 3 T or 7 T. Therefore, our calibration was still acceptable at pH 7.0. Yet, even at 9.4 T, measurements at the higher end of the pH range are less precise than at lower pH. A recent study has addressed this problem by using the Bloch equations to fit CEST spectra at 3 T and 7 T, but was not investigated at 9.4 T [21].

The slope of CEST-pH calibration was lower in our study at 9.4 T compared with previous studies at 3 T [22], 4.7 T [23, 24], and 7 T [25]. This different slope of the calibration curve is intuitive. At 7 T, the loss of CEST from the amide proton at 5.6 ppm is particularly noticeable at pH 7.0 and higher, while the loss of CEST from the amides at 4.2 ppm is noticeable at pH 7.5 and higher. This difference in CEST amplitude is also apparent at lower field strengths. For comparison, a 9.4 T magnetic field strength generates a larger chemical shift difference in Hz, retaining similar CEST signal amplitudes at higher pH values for both amides resonating at 5.6 and 4.2 ppm. Therefore, a ratiometric-based analysis has a lower slope for the CEST-pH calibration at 9.4 T. Nevertheless, the linear CEST-pH relationship (Fig. 1f) and the reproducible pH values reported *in vivo* (Fig. 6a, b) indicate that our pH measurements were valid at 9.4 T.

A recent study reported a different ratiometric approach to measuring pH with CEST MRI that used a ratio of the CEST signal at 5.6 ppm acquired at 2 μT saturation power and 4.2 ppm at 1 μT [26]. The higher saturation power of 2 μT generated a stronger CEST signal at 5.6 ppm than at 1.0 μT, which was especially useful at the 4.7 T magnetic field strength used in that study. We conducted our studies at 9.4 T and observed a measurable signal at 5.6 ppm, so that the dual-power approach was less necessary for our studies. Also, our single-power approach required half of the data acquisition time compared with the dual-power approach, which is advantageous for reducing preclinical scan time for our studies.

We performed our *in vitro* pH calibration studies at an average temperature of 33.5 °C, but we maintained an average temperature of 34.5 °C during our mouse experiments. Although there should be a match between the temperatures of two experiments, we do not expect a significant error in the measured pH values because our ratiometric analysis reduces the effects of many types of errors. In this case, a higher mouse temperature would increase the chemical shift rates of both agents, and the ratio tends to cancel effects that change both CEST signals [25].

Typically, acidoCEST measurements of tumors are performed using a steady infusion of the CA [5, 6, 9, 25]. Such steady-state infusion may not be necessary for kidney pH measurements. Considering the pharmacokinetics of the kidney, a slow infusion would be quickly filtered and have less benefit than for tumors that are typically poorly perfused [32]. Therefore, we used a relatively slow bolus to inject the CA and did not use a post-injection infusion.

The total acquisition time for our anatomical, ASL, and acidoCEST MRI protocol was 60 min with respiratory gating. This time may raise concerns that the concentration of iopamidol was changing during the entire acidoCEST MRI scan session, and the two CEST signals may have been measured with different CA concentrations, and therefore the comparison of two CEST signals to measure pH may depend on concentration. However, the key timing for the pH measurement is the time to acquire the CEST MR images around 5.6 ppm and 4.2 ppm for a single CEST spectrum which define the shape and height of the CEST peaks that are used for Lorentzian line fitting [8]. The timing for acquiring CEST MR images in this small ppm range is on the order of tens of seconds. Importantly, the change in CA concentration within tens of seconds should be negligible based on our flow measurements. Even more importantly, acidoCEST MRI may provide the most accurate measurements for the impaired kidney that has even lower flow and perfusion, providing an excellent imaging methodology for this specific pathology [27].

Our results demonstrated that iopamidol injection and MRI measurements had a clear influence on the subsequent kinetics of IRDye clearance measured by MSOT in both the renal cortex and pelvic/medulla regions. The mean cortex decay time was significantly elevated when MR measurements were carried out before MSOT measurements (C:P AUC measurements were not significantly elevated). Iopamidol has a viscosity of ~ 7 mm^2^/s and an osmolality of 796 mosmol/kg H_2_O [10, 19]. This markedly higher viscosity of iopamidol compared with blood plasma may lead to enrichment of iopamidol in the renal tubules where water is reabsorbed, resulting in increased tubular fluid viscosity, hindered glomerular filtration, and reduced medullary blood perfusion [10, 19]. Therefore, renal measurements using MSOT and ASL MRI should be performed prior to the administration of iopamidol for acidoCEST MRI.

For comparison, MRI parameter values were similar regardless of the order of MRI and MSOT scans. However, larger variabilities were observed among the pH measurements between individual animals from both the renal cortex and pelvis when MSOT was performed prior to the MRI measurements as demonstrated by the coefficients of variation (Table 1). We speculate that the higher standard deviation in pH and perfusion could be due to the variation in the volume of residual IRDye before the MRI measurements. Clearance kinetics of IRDye from previous studies indicate that 48 h may be required for this dye to be completely cleared through kidneys [28], which may impede any test-retest studies to evaluate the repeatability of the measurements during the same study.

Our group has previously reported the utility of MSOT for assessing renal function of healthy mice and those with chronic kidney disease [16]. In that study, the Tmax delay (time between the Tmax in the cortex and the Tmax in the pelvis) as well as the exponential decay time of IRDye from the renal cortex were used as measures of kidney function. In the present study, we used the cortex decay time and C:P AUC to assess kidney function. The C:P AUC was observed to be a more appropriate parameter in this study because it was not susceptible to large changes in the shape of the uptake-clearance curve [18].

Testing the reproducibility of pH, perfusion, and kidney function measurements on the same animals on days 1 and 4 revealed slight but significant differences in blood flow from the kidney cortex and pelvis. While the cortical blood flow increased on day 4, the pelvic values decreased by day 4. The pH values were similar to the recently reported values for kidney in the range of pH 6.3–7.0 [6]. Previously, a study using a T1 MRI contrast agent to measure renal pH in mice found that the kidney pH ranged from 6.6 to 7.4 and that pH of the renal cortex was consistently higher than that of the pelvis [29] and our studies are in line with these observations, although we realize that this is a considerably large range for the kidney which has a very tight and active homeostatic mechanism to maintain the pH. It is possible that the larger range in pH values is due to a relatively large ROI used to depict the entire kidney cortex or pelvis and that there are differences within these ROIs that may have averaged out. Due to the limited spatial resolution and the number of pixels that the acidoCEST data could be fitted, we used such larger ROIs. Future studies with higher resolution 3D acidoCEST measurements may allow assessment of the intra-cortex kidney differences better. Although respiratory gating was used during blood flow assessments, FAIR-based perfusion measurements are known to be susceptible to motion artifacts. The fact that the cortex and pelvic values changed in opposite directions support the explanation that these values may in fact be affected by motion and that these results should be regarded with caution considering the susceptibility of ASL methods to motion-induced artifacts. Additional limitations of the study include a relatively small sample size of three mice in each group to assess the dependence of CA on MRI or MSOT experiments. It is also possible that due to the relatively high viscosity of iopamidol, the kidney perfusion changed. Unfortunately, we did not measure kidney perfusion using the FAIR-EPI method before and after injection of iopamidol as the main goal of our study was to demonstrate the feasibility of MSOT and MRI to assess kidney function. Future studies are warranted to evaluate the effect of iopamidol on kidney perfusion.

## Conclusions

In conclusion, we have demonstrated that consecutive MRI measurements of renal pH and perfusion, and MSOT measurement of renal clearance, are feasible with reproducible quantitative values. Both the MSOT and MRI techniques were complimentary and provided more detailed information about pH, renal blood flow, and clearance. The MSOT studies should be performed before administration of iopamidol for acidoCEST MRI. These results establish a dual modality method with MSOT and MRI to support future studies that evaluate acute and chronic renal injury models.
